# Naso-Ethmoidal Schwannoma: From Pathology to Surgical Strategies

**DOI:** 10.3390/cancers17071068

**Published:** 2025-03-22

**Authors:** Sergio Corvino, Oreste de Divitiis, Giuseppe Corazzelli, Jacopo Berardinelli, Adriana Iuliano, Chiara Di Domenico, Vittoria Lanni, Roberto Altieri, Diego Strianese, Andrea Elefante, Giuseppe Mariniello

**Affiliations:** 1 Department of Neurosciences and Reproductive and Odontostomatological Sciences, Division of Neurosurgery, School of Medicine, University of Naples “Federico II”, Via S. Pansini 5, 80131 Naples, Italy; oreste.dedivitiis@unina.it (O.d.D.); iacopobe96@gmail.com (J.B.); didomenico.chiara.nch@gmail.com (C.D.D.); giumarin@unina.it (G.M.); 2 Neurosurgery Department, Santa Maria delle Grazie Hospital, ASL Napoli 2 Nord, 80078 Naples, Italy; giucoraz@gmail.com; 3 Department of Neurosciences and Reproductive and Odontostomatological Sciences, Division of Ophthalmology, School of Medicine, University of Naples “Federico II”, 80131 Naples, Italy; adrianaiuliano@yahoo.it (A.I.); vittoria.lanni@unina.it (V.L.); diego.strianese@unina.it (D.S.); 4 Multidisciplinary Department of Medical-Surgical and Dental Specialties, University of Campania “Luigi Vanvitelli”, 80131 Naples, Italy; roberto.altieri.87@gmail.com; 5 Department of Advanced Biomedical Sciences, Division of Neuroradiology, School of Medicine, University of Naples “Federico II”, 80131 Naples, Italy; aelefant@unina.it

**Keywords:** schwannoma, endoscopic endonasal approach, transorbital approach, head tumors

## Abstract

**Background:** Naso-ethmoidal schwannoma is a rare slow-growing tumor arising from the Schwann cells of the sinonasal tract. This study discusses the evolution of surgical approaches to naso-ethmoidal schwannomas with respect to tumor growth and recent advances in minimally invasive techniques. **Materials and Methods:** A comprehensive literature review on Embase online electronic database on benign naso-ethmoidal schwannoma was performed. Demographic, clinical, neuroradiological, pathological, and surgical factors were analyzed and discussed. **Results:** Twenty-five cases met the inclusion criteria. No predilection for gender was evident. The mean age at diagnosis was 40.2 years old. Nasal obstruction was the most common presenting symptom (64%), followed by headache (60%), hypo-anosmia (24%), and visual impairment (24%). Skull base and orbital involvement were registered in 64% and 16% of cases, respectively. Surgery is the unique curative treatment, with the endoscopic endonasal approach which plays the leading role (44%). Gross total resection was possible in all cases and was associated with no recurrence. The perioperative complication rate was 32% and mainly consisted of cerebrospinal fluid leakage. The mean time for treatment was 21 months. All patients were alive at last follow up. **Conclusion:** Surgery is the only curative treatment for naso-ethmoidal schwannomas, with the main goal to relief clinical manifestations. The endoscopic endonasal route represents the master approach for lesions confined to the midline. Transcranial and transorbital approaches play a complementary role when large intracranial extension and orbital involvement occur, respectively.

## Simple Summary

Schwannomas are benign nerve sheath tumors that grow slowly and can arise anywhere in the body, but only 4% arise in the sinonasal tract. Although benign, their close proximity to important structures such as the orbit and brain can lead to debilitating, potentially irreversible neurological and ophthalmological deficits. Therefore, a timely diagnosis and appropriate management are mandatory. Curative treatment is surgery, with the endoscopic endonasal approach representing the main surgical route. Transcranial and transorbital approaches play a complementary role when large intracranial extension and orbital involvement occur, respectively. This study discusses the evolution of surgical approaches to naso-ethmoidal schwannomas with respect of tumor growth and recent advances in minimally invasive techniques.

## 1. Introduction

Schwannomas are slow-growing benign nerve sheath tumors derived from Schwann cells that contribute to axonal myelination in the peripheral nervous system. Theoretically they can originate from anywhere in the body [1,2,3,4,5,6,7,8], but up to 45% occur in the head and neck, of which the most common location is the eight cranial nerve, while 4% affect the sinonasal tract [9,10]. Ethmoid schwannomas often present with mass effect symptoms, such as nasal obstruction, epistaxis, headache, and visual disturbances [11]. These are not specific and can mimic several and heterogeneous sinonasal diseases, with different natural history, as such as fibrosarcoma, leiomyosarcoma, solitary fibrosus tumor, fibromatosis, and biphenotypic sarcoma [12,13,14].

To date, the main factors driving tumorigenesis and progression of schwannomas are yet to be fully understood [15]. However, the biallelic inactivation of NF2 [16] accounting for loss-of-function mutations in merlin, is the primum movens that triggers the initial tumorigenic transformation of Schwann cells. Merlin is a protein involved in intercellular adhesion, and when mutated, it makes Schwann cells more vulnerable to the injury. In this context, after nerve damage, the Schwann cells undergo dedifferentiation and neoplastic transformation. This supports the etiopathogenetic hypothesis that the origin schwannoma could result from the repair process subsequent to a nerve injury [17,18]. Nevertheless, NF2 mutations are not present in all schwannomas, therefore, other different genetic alterations have been considered in the tumorigenesis, including the SH3PXD2A::HTRA1 fusion gene, SOX10 indel mutation, and VGLL-fusions. In addition, recent studies have clarified the role of the tumor microenvironment in the schwannoma development. However, to date, the exact biomolecular anomalies at the origin of schwannomas is still a matter of debate [15,19].

Despite its benign biological behavior, characterized by slow growth and unencapsulated and not-infiltrative pattern of growth, no tendency to recur after complete resection and very rare malignant transformation, because of its anatomical origin at the upper airways and near to vulnerable structures, like the orbital content and the brain, it may account for severe and potentially irreversible neurological–ophthalmological deficits. Therefore, prompt diagnosis and adequate management are mandatory.

In this setting, the aim of the present study was to discuss the evolution of the rationale, as well as the surgical approach selection, behind the strategy of treatment of naso-ethmoidal schwannomas (NES) in tandem with the better knowledge of the clinical course of pathology and the advances of minimally invasive surgery, through a retrospective analysis from a comprehensive literature review.

## 2. Methods

**Figure 1:**
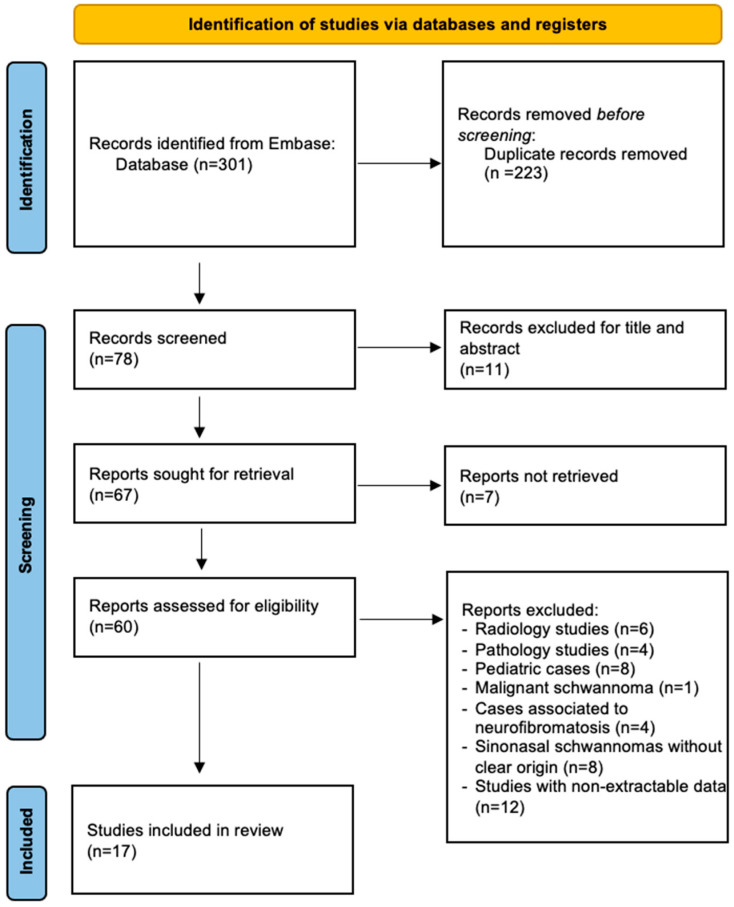
PRIMA (Preferred Reporting Items for Systematic Reviews and Meta-Analysis) flow chart showing the methods for the selection of the studies included in the review. Source: Page M.J. et al. [20]. This work is licensed under CC BY 4.0. To view a copy of this license, visit https://creativecommons.org/licenses/by/4.0/.

A systematic review of well-differentiated naso-ethmoidal schwannomas was performed in Embase database, according to Preferred Reporting Items for Systematic Reviews and Meta-Analysis (PRISMA) guidelines [20], by using the following key sentences: (“schwannoma” OR “neurilemmoma” OR “neurilemoma”), (“ethmoid sinus” OR “ethmoidal sinus”), (“nasal cavity” OR “nasal cavity”), (“sinonasal tract” OR “sinonasal AND tract”), “orbit”. They were combined as follows: (“schwannoma” OR “neurilemmoma” OR “neurilemoma”) AND (“ethmoid sinus” OR “ethmoidal sinus”), (“schwannoma” OR “neurilemmoma” OR “neurilemoma”) AND (“nasal cavity” OR “nasal cavity”), (“schwannoma” OR “neurilemmoma” OR “neurilemoma”) AND (“sinonasal tract” OR “sinonasal AND tract”), (“schwannoma” OR “neurilemmoma” OR “neurilemoma”) AND (“ethmoid sinus” OR “ethmoidal sinus”) AND (“nasal cavity” OR “nasal cavity”), (“schwannoma” OR “neurilemmoma” OR “neurilemoma”) AND (“ethmoid sinus” OR “ethmoidal sinus”) AND (“nasal cavity” OR “nasal cavity”) AND “orbit”. No restrictions on publication year were applied to ensure a comprehensive collection of reported cases. After duplicate removal and the screening for title and abstract, the remaining studies were carefully selected by two authors (S.C. and C.D.D.) and enrolled in this systematic review according to the inclusion and exclusion criteria (Figure 1).

The inclusion criteria encompassed surgical series, reviews, and case reports in English focusing on benign ethmoidal schwannomas, confirmed by immunohistochemical diagnosis and explicitly localized to the ethmoid. Studies were required to report relevant clinical and surgical data, including those analyzed in this review (patient sex and age, presenting symptoms and signs, anatomical origin and growth pattern, time to treatment, type of treatment, surgical approach, extent of resection, perioperative complications, recurrence, and overall survival).

Exclusion criteria included studies involving pediatric patients (<18 years), as schwannomas in children may differ in presentation and management. Duplicate publications were excluded to prevent data redundancy. Studies on schwannomas in other locations, including those of the sinonasal tract without a clearly defined ethmoidal origin, were omitted to ensure a homogeneous cohort. Radiology studies, literature reviews, and case series lacking extractable clinical and surgical data were excluded, as they did not provide sufficient information for analysis. Finally, cases associated with von Recklinghausen’s disease were excluded to avoid confounding factors related to the underlying genetic disorder.

### Statistical Analysis

Categorical and qualitative data were evaluated using the Shapiro–Wilk test for normality, with a significance level of *p* < 0.05. Data were aggregated in Microsoft Excel (version 14.2.5), and GraphPad software (version 10.2.2) was used to perform the analysis.

## 3. Results

A comprehensive systematic literature review disclosed 83 studies concerning schwannomas of the ethmoid sinus, 173 on schwannomas affecting the nasal cavities, 45 studies about schwannomas of the sinonasal tract. After duplicates removal, screening full texts of the marked studies included according to the inclusion criteria, 17 studies reporting 25 cases were identified.

All patients’ data are separately reported in Table 1 and Table 2 and summarized in Table 3 and Table 4.

- Patients and pathology features (Table 3)

The gender distribution was almost even, 52% of patients were female, and 48% were male, with median age of 40.2 years (range 22–75 y.o.). The presenting symptoms were mainly represented by nasal obstruction (n. = 16/25, 64%), followed by headache (n. = 15/25, 60%), hypo-anosmia (n. = 6/25, 24%), and ocular impairment (n. = 6/25, 24%).

From the anatomical site of origin represented by ethmoid sinus, the lesion extended to the intracranial space in 64% of cases (n. = 16/25) and into the orbital cavity in 16% (n. = 4/25).

The time to treatment was reported in nine among the 25 cases (36%) and the mean value was 21 months.

- Treatment and outcome findings (Table 4)

The type of treatment administered was reported in all studies. Surgery was performed in all cases as single treatment (25/25, 100%).

The description of the type of surgical approach selected was reported in 25 out of 25 procedures (100%). The most adopted surgical corridor was the endoscopic endonasal route (n. = 11/25, 44%), followed by the isolated microsurgical transcranial approach as frontal craniotomy (n. = 7/25, 28%), the combined approaches (n. = 5/25, 20%), and finally the functional endoscopy sinus surgery (FESS) (n. = 2/25, 8%).

The extent of tumor resection was reported in all cases (100%) and gross total resection (GTR) was always accomplished.

Only in nineteen cases (76%) was the date concerning perioperative complications reported. They occurred in six patients (32%) and consisted in transient CSF leak (5), in one case associated with meningitis and frontal abscess, and hematoma and enophthalmos (1).

Data on recurrence rates were reported in 21 cases of the overall sample (84%) and no cases of recurrences was reported. Finally, the follow-up was 15.33 months (±18.67).

All patients were alive at last follow-up.

## 4. Discussion

Adachi et al. [38] include NES among subfrontal schwannomas, which are classified as the schwannoma of the “olfactory site” (olfactory groove or cribriform plate) and “other than olfactory site” schwannomas (from nonolfactory sites). Later, Yoneoka et al. [39] revised this classification identifying four types: sub-frontal (olfactory groove), naso-ethmoidal (sinonasal), frontoethmoidal (primarily intracranial with extension into the paranasal sinuses), or ethmo-frontal (primarily sinonasal with intracranial extension).

The origin of the naso-ethmoidal schwannoma in a cavity, along with its slow growth, account for the long silent course of the disease (21 months as the mean time from clinical symptoms onset to diagnosis), until the lesion reaches large sizes and has a mass effect on the near anatomical structures leading to nasal obstruction, headaches, hypo-anosmia, and ocular impairment.

Some uncertain pathologic findings, like the exact structure of origin and the pattern of growth of the tumor, characterize the NES.

The major involvement of the ethmoid bone and nasal cavity by schwannomas has been associated with the more abundant and complex innervations of these anatomical regions [40]. In particular, they are believed to arise from the ophthalmic and maxillary branches of the trigeminal nerve or from autonomic nerves to the septal vessels and mucosa. Nevertheless, because of the small sizes of the nerves of origin, it is almost never possible to distinguish an attached nerve during surgery [41,42].

In addition, some authors describe NES as encapsulated lesions with well-defined margins, and vice versa, most suggest that naso-ethmoidal schwannomas, like those arising in nervous parenchyma, skin, viscera, and bone, are not encapsuled [42,43,44,45,46]. They assume that this finding is due to the development of these tumors from sinonasal mucosa autonomic nervous system fibers, which are devoid of perineurial cells [44].

As for those affecting the spinal canal with extraforaminal extension [5], characterized by a dumbbell-shape pattern of growth, schwannomas arising from the ventral side of the ethmoid bone can assume a tubular conformation due to the lamellae of the ethmoid which mold the tumor shape during its growth toward the nasal cavity, frequently associated with pressure remodeling/erosion of the adjacent bone leading to lesion extension beyond the original site to the anterior skull base (64%) and/or orbital cavity (16%).

However, this multicompartmental involvement, together with the radiological appearances of local bone invasion and histological features of tumor un-encapsulation, does not correlate with malignant potential, and therefore does not justify unnecessary over-treatment. In this setting, several authors usually perform and recommend a preoperative biopsy to rule out the possibility of malignant lesions such as plasmacytoma or malignant schwannoma which could radiologically mimic benign schwannomas [47].

Over the years, we witnessed to a change in the paradigm of treatment concerning many different neoplastic lesions affecting functional areas in neurosurgery, shifting from the need of a maximal tumor resection toward the preservation of a patient’s good quality of life. For example, in meningiomas surgery, which, despite the Simpson grade I, represents the goal of treatment, is not always achievable, like for some lesions affecting critical areas, such as the spheno-orbital region. Also, subtotal resection followed by adjuvant treatment or re-surgery at the onset of new symptoms represent valid alternative options [48,49]. Also, in low-grade gliomas in eloquent areas [50], in which taking the advantages of the postoperative cerebral plasticity, the first tumor resection can be incomplete to preserve the anatomy and function of the affected area. Meanwhile, postoperative functional remapping will allow a more extensive resection while preserving brain functions in a multistage surgical approach [51,52]. Further, in large vestibular schwannomas, where a subtotal resection followed by stereotactic radiosurgery is justified to avoid the iatrogenic risk of damage to facial nerves, while reducing the mass effect and improve clinical symptoms/arrest or prevent deterioration of auditory function [53].

In line with this principle, we consider that the goals of treatment of naso-ethmoidal schwannomas are to (i) ensure upper airway patency, (ii) reduce the mass effect on the adjacent structures, and (iii) prevent associated complications.

For these purposes, surgical resection represents the only curative modality of treatment, and the decision of a more or less aggressive strategy in terms of extent of resection, as well as the selection of the surgical approach, must be tailored case by case according to patients and pathological features during preoperative planning. In this scenario, maximal safe tumor resection represents the standard of care and should be attempted when no surgical risk to vulnerable functional neurovascular structures could occur. Conversely, despite the fact that subtotal resection is associated with higher recurrence rates, considering the benign nature of the lesion, characterized by the slow-growth, not infiltrative spreading pattern, no tendency to malignant transformation nor to metastasize [39], also a more conservative strategy, including safe tumor debulking aiming to symptoms and signs relief, followed by eventual re-surgery when tumor regrows/recurs, is a valid alternative option [10].

The main issue affecting the surgical strategy includes the extension of the lesion to the adjacent anatomical structures. The ethmoid bone is deep-seated in the midline skull base and represents a crossroads between different anatomical compartments which enclose highly functional structures, such as the intracranial space superiorly, nasal cavity inferiorly and orbits bilaterally.

Traditional external approaches include lateral rhinotomy, Caldwell–Luc with or without Denker extension or midface degloving, and the endoscopic endonasal approach (EEA).

Over the last decades, the endoscopic endonasal route has become the master approach for ventral midline skull base lesions like NES, and for those with intracranial spreading from the crista galli to the odontoid [54,55]. Srinivasan and Klossek described the endonasal endoscopic approach for the removal of a benign neurilemmoma [56,57]. This corridor provides a straight and short trajectory to the target, through a favorable angle of strategy, with low morbidity, short hospital stay, avoiding craniotomy and manipulation of nervous structures. Therefore, it is the standard of care for NSE confined to the midline, limited to the nasal cavity or with extension to the anterior skull base or to the medial orbital compartment, as single or combined, in single or multistage approach, for tumor biopsy or resection. The most common intra-perioperative complication is the CSF leak; however, the continuous refinements of the reconstruction techniques has led to a significant decrease in of incidence [58,59,60].

A valid alternative for lesions confined to the nasal cavity and with extension to the medial orbital cavity through the lamina papyracea, is represented by the medial transorbital approach [61,62]. During the last two decades, endoscopic transorbital approaches have become very popular among neurosurgeons for addressing pathologies affecting the paramedian anterior and middle skull base [63,64,65,66,67,68,69]. The medial transorbital approach avoids a Weber–Ferguson incision, while allowing for an accurate assessment of the medial orbital wall and periorbita for tumor invasion using direct intraoperative feedback. Further benefits include hemostasis through the cauterization of the anterior and posterior ethmoid arteries. The risks include diplopia, lacrimal injury, medial canthal tendon injury and scarring. Scarring and diplopia can be avoided by keeping the incision medial to the conjunctiva of the globe, and adequate retraction should allow us to avoid the lacrimal sac [62].

For lesions with large intracranial involvement, especially with lateral extension in the coronal plane, a transcranial approach through bifrontal craniotomy represented the most selected approach.

Open transcranial and endoscopic endonasal and transorbital surgical routes can be selected as single or multiportal combined approaches, according to the pathology and patient features, providing a 360° circumferential access to the tumor affecting the naso-ethmoidal region.

### Limitation of the Study

This study is limited by the retrospective nature and small size and heterogeneity of the data derived predominantly from case reports. Additionally, the variability in the reporting of clinical details across studies limited the possibility of analyzing potential confounders, which should be considered when interpreting the findings.

## 5. Conclusions

Naso-ethmoidal schwannomas are rare benign tumors with a tendency to invade the intracranial space and orbital cavity through pressure remodeling/erosion of the adjacent bony structures. Surgery is the only curative treatment with the aim to relieve symptoms and signs. The endoscopic endonasal approach represents the master approach for midline lesions limited to the nasal cavity with or without median anterior skull base and/or medial orbital compartment involvement. The medial transorbital presents the advantage to directly assess the microsurgical relationship between the tumor and orbit content. Transcranial approaches are reserved for lesions with large intracranial extension.

## Figures and Tables

**Table 1:** Demographic, clinical, radiological and pathological data of 25 cases of naso-ethmoidal schwannomas.

	Authors/Year	Num. of Cases	Sex, Age (Years)	Presenting Symptoms	Anatomical Origin	Intracranial Extension	Orbit Involvement
1	Zovickian et al. [21] 1986	1	M, 40	Nasal obstruction, headache	ES	Yes/ACF	Not
2	Enion et al. [22] 1991	1	M, 28	Headache, nausea, vomiting, epilepsy, blurred vision	ES	Yes/ACF	Not
3	Bavetta et al. [23] 1993	1	M, 41	Anosmia, nasal obstruction, blurred vision, diplopia, proptosis	ES-SS-FS	Yes/ACF	Yes
4	Gatscher et al. [24] 1998	1	F,50	Anosmia, headache, visual disfunction	ES	Yes	Not
5	Sharma et al. [25] 1998	1	M, 35	Anosmia, nasal obstruction, epistaxis, epilepsy	ES	Yes/ACF	Not
6	Siqueira et al. [26] 2001	1	F, 40	Anosmia, headache	ES	Yes/ACF	Not
7	Pasquini et al. [27] 2002	1	F, 75	Nasal obstruction	ES	None	Not
8	George et al. [28] 2009	1	F, 27	Blurred vision, headache	ES	Yes	Yes
9	Suh et al. [29] 2011	3	M, 51	Nasal obstruction, headache	ES	None	Not
			F, 68	Nasal obstruction, headache	ES	None	Not
			F, 49	Headache	ES	None	Not
10	Blake et al. [30] 2014	1	M, 62	Hyposmia Hypogeusia	ES	Yes/ACF	Not
11	Zhou et al. [31] 2015	3	M, 32,	Nasal obstruction, hyposmia	L MS/ES/SS/NC; NS	None	Not
			F, 57	Nasal obstruction	R. MS/ES/SS/NC		
			F, 42	Nasal obstruction, headache	R. ES/NC;		
12	Hong et al. [32] 2016	1	M, 24	Asymptomatic	ES	Yes/ACF	Not
13	Eichberg et al. [33] 2017	1	M, 41	Anosmia, headache	ES	Yes/ACF	Not
14	Narang et al. [34] 2019	1	F, 33	L. proptosis, epistaxis	ES	Yes	Yes
15	Rogister et al. [35] 2021	1	M. 72	Right orbital cellulitis	ES	None	Yes
16	Brahmbhatt et al. [36] 2023	5	3F, 2M (mean age 48 yrs)	Headache, Fullness Nasal Obstruction Dizziness, Nonspecific Neurologic	5 ES	5 Yes	5 Not
17	Hachicha et al. [37] 2024	1	F, 22	Nasal obstruction, hyposmia, epistaxis	ES-SS	None	Not

M: Male, F: Female, n.a.: not available; l: left; ACF: Anterior Cranial Fossa; mo.: months; ES: Ethmoid Sinus: FS: Frontal Sinus; SS: Sphenoid Sinus; MS: Maxillary Sinus; NC: Nasal Cavity.

**Table 2:** Treatment and outcome data of 25 cases of naso-ethmoidal schwannomas.

	Authors/ Year	Num. of Cases	Time to Treatment	Type of Treatment	Type of Surgical Approach	EOR	Reconstruction	Peri-Post Operative Complications	Recurrence	Status at Last f.u.
1	Zovickian et al. [21] 1986	1	n.a.	S	FC + EEA	GTR	n.a.	None	n.a.	n.a.
2	Enion et al. [22] 1991	1	9 months	S	BFC	GTR	graft of lyophilized dura.	CSF leak	n.a.	Alive 3 mo
3	Bavetta et al. [23] 1993	1	36 months	Biopsy- S	FC + EEA	GTR	split skin graft, pericranial flap and lyodura	Enophthalmos, hematoma.	n.a.	n.a.
4	Gatscher et al. [24] 1998	1	36 months	S	BFC	GTR	n.a.	n.a.	n.a.	n.a.
5	Sharma et al. [25] 1998	1	60 months	S	BFC	GTR	vascularised pericranial flap.	CSF leak	None	Alive 6 mo
6	Siqueira et al. [26] 2001	1	36 months	S	BFC + Lateral rhinotomy	GTR	n.a.	None	None	Alive 5 yrs
7	Pasquini et al. [27] 2002	1	6 months	S	EEA	GTR	n.a.	n.a.	None	Alive 55 mo.
8	George et al. [28] 2009	1	6 months	S	FC	GTR	Tisseel Gortex	CSF leak	None	Alive 3 mo.
9	Suh et al. [29] 2011	3	n.a.	S	EEA	GTR	n.a.	n.a.	None	Alive 13 mo
			n.a.	S	EEA	GTR	n.a.	n.a.	None	Alive 53 mo.
			n.a.	S	EEA	GTR	n.a.	n.a.	None	Alive 6 mo
10	Blake et al. [30] 2014	1	4 months	biopsy- S	EEA	GTR	fascia lata, dermal allograft, vascularized nasoseptal flap	None	None	Alive 5 mo.
11	Zhou et al. [31] 2015	3	n.a.	3 S	1 FESS	3 GTR	n.a.	None	None	3 Alive (mean 5 yrs)
					1 FESS					
					1 FESS + LR					
12	Hong et al. [32] 2016	1	Incidental	S	BFC	GTR	Autologous muscle graft, free pericranial graft, and synthetic dura substitute.	CSF leak, meningitis, frontal abscess	n.a.	n.a.
13	Eichberg et al. [33] 2017	1	Several months	S	BFC	GTR	Pericranial flap, watertight dural closure	None	None	Alive 1 yrs
14	Narang et al. [34] 2019	1	3 months	S	BFC + EEA	GTR	Pericranial flap, abdominal free fat graft, mucosa, fibrin glue	None	None	Alive 7 mo.
15	Rogister et al. [35] 2021	1	n.a.	S	EEA	GTR	n.a.	n.a.	None	Alive 8 mo.
16	Brahmbhatt et al. [36] 2023	5	n.a.	5 S	4 EEA 1 FC	5 GTR	n.a.	1 CSF leak 4 None	5 None	n.a.
17	Hachicha et al. [37] 2024	1	n.a.	biopsy- S	EEA	GTR	n.a.	None	None	Alive 20 mo.

n.a.: not available; GTR: Gross Total Resection; STR: Sub Total Resection; S: Surgery; RT: Radiotherapy; BFC: Bifrontal Craniotomy; EEA: Endoscopic Endonasal Approach.

**Table 3:** Summarized available demographic, clinical, neuroradiological, and pathological data of 25 cases of naso-ethmoidal schwannomas.

Covariates	Overall Sample 25 (%)	Statistical Analysis (*p* Value)
Demographic and clinical data		
Sex - F - M	13/25 (52%) 12/25 (48%)	*p* = 1.0
Age range (median)	22–75 years (40.2 y.o.)	S-W = 0.93; *p* = 0.23
Main presenting symptoms - nasal obstruction - headache - anosmia/hyposmia - visual impairment	16/25 (64%) 15/25 (60%) 6/25 (24%) 6/25 (24%)	*p* = 0.57
Time to treatment (mean ± SD)	9/25 * (36%) 21 mo.	S-W = 0.847; *p* = 0.09
Radiological data		
Orbital involvement - Yes - Not	4/25 (16%) 21/25 (84%)	*p* < 0.01
Skull Base involvement - Yes - Not	16/25 (64%) 9/25 (36%)	*p* = 0.89

* available data.

**Table 4:** Summarized available treatment and outcome data of 25 cases of naso-ethmoidal schwannomas.

Covariates	Overall Sample 25 (%)	Statistical Analysis (*p* Value)
Treatment Data		
Type of treatment - S	25/25 (100%)	
Type of surgical approach - EEA - BFC - FESS - Combined	11/25 (44%) 7/25 (28%) 2/25 (8%) 5/25 (20%)	*p* = 0.12
EOR - GTR	25/25 (100%)	
Peri and postop complications - Yes - None	19/25 * (76%) 6/19 (32%) 13/19 (68%)	*p* = 0.06
Outcome		
Recurrence - Yes - Not	21/25 * (84%) 0/21(0%) 21/21 (100%)	-
Status - Alive	16/25 * (64%) 16/16 (100%)	-
Follow-up (mean ± SD)	15.33 (±18.67)	S-W = 0.65; *p* < 0.01

* available data.

## Data Availability

Data of the current original research are available from the corresponding author on reasonable request.
